# A Late Presentation of COVID-19 Vaccine-Induced Myocarditis

**DOI:** 10.7759/cureus.17890

**Published:** 2021-09-11

**Authors:** Nitesh Gautam, Prachi Saluja, Marat Fudim, Kedar Jambhekar, Tarun Pandey, Subhi Al'Aref

**Affiliations:** 1 Internal Medicine, University of Arkansas for Medical Sciences, Little Rock, USA; 2 Cardiology, Duke University Medical Center, Durham, USA; 3 Radiology, University of Arkansas for Medical Sciences, Little Rock, USA; 4 Cardiology, University of Arkansas for Medical Sciences, Little Rock, USA

**Keywords:** covid-19 vaccine complication, atypical chest pain, vaccine, post vaccination myocarditis, covid 19

## Abstract

With the introduction of the coronavirus disease 2019 (COVID-19) mRNA vaccines, the incidence of severe infection has significantly decreased. While the vaccines have been shown to be effective and safe, there have been few case reports of acute myocarditis within 3-5 days following the second dose of the vaccine. We report a case of an elderly man who presented with acute-onset chest pain after three months of receiving the second dose of the mRNA vaccine. He was found to have acute myocarditis on cardiac magnetic resonance imaging (CMRI), which was attributed to exposure to the COVID-19 vaccine in the absence of any other risk factors. Our patient demonstrated quick resolution of symptoms and was discharged within 72 hours. We review the literature and summarize published case reports on COVID-19 vaccine-associated myocarditis. The present case report provides new evidence regarding the possible subacute presentation of myocarditis post-COVID-19 vaccine, and further highlights the favorable outcome in this newly described clinical entity.

## Introduction

The coronavirus disease 2019 (COVID-19) pandemic has incurred significant morbidity and mortality across the globe [1]. The cardiovascular manifestations of an acute COVID 19 infection include acute coronary syndrome, as well as myocarditic inflammation [2]. With the development and recent introduction of COVID-19 vaccines, the incidence of severe infection has significantly decreased [1]. While the COVID-19 vaccine has been proven to be effective and safe across various populations, there have been few case reports of acute myocarditis within 3-5 days following the second dose of the COVID-19 vaccine [1,3-9]. According to the Centers for Disease Control and Prevention (CDC), >1000 reports of vaccine-induced myopericarditis have been recorded by the Vaccine Adverse Event Reporting System [10]. We report a case of an elderly man who presented with acute-onset chest pain three months after receiving the second dose of the mRNA vaccine. He was found to have acute myocarditis on cardiac magnetic resonance imaging (CMRI), which was attributed to exposure to the COVID-19 vaccine. To the best of our knowledge, this is a rare case of subacute vaccine-induced myocarditis, which has not been described in the literature so far.

## Case presentation

**Figure 1:**
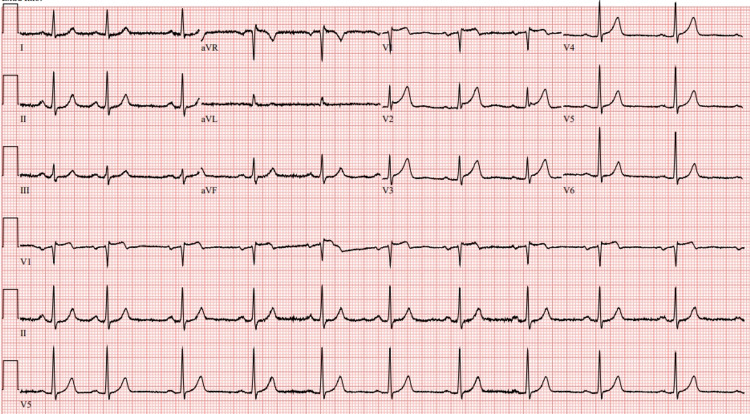
12-lead electrocardiogram for the patient demonstrating <1 mm ST elevation in the anterior leads.

A 66-year-old Caucasian man with a prior history of hypertension, type II diabetes mellitus, and hyperlipidemia presented with sudden onset of crushing substernal chest discomfort. The pain occurred 4 hours prior to the presentation while he was lying in bed. He also experienced diaphoresis and two episodes of emesis. Chest pain was not associated with any cough or shortness of breath. The patient did not report any recent history of respiratory illnesses. He had received the second dose of the BNT162b2 (Pfizer-BioNTech) vaccine three months prior to presentation, without any immediate adverse events following the vaccination. In the emergency room, he was afebrile with an elevated blood pressure of 167/77 mmHg, heart rate of 63/min, breathing 22/min, and saturating well on room air. Physical examination was remarkable for an anxious appearing man with a benign cardiopulmonary exam. Initial labs showed mild elevation of erythrocyte sedimentation rate (ESR; 40 mm/hour) with a normal C-reactive protein (CRP). Complete blood count (CBC) and basic metabolic panel (BMP) were unremarkable, while the initial highly sensitive troponin-I was negative. ECG showed <1-mm ST elevation in the anterior leads (Figure 1). 

**Figure 2:**
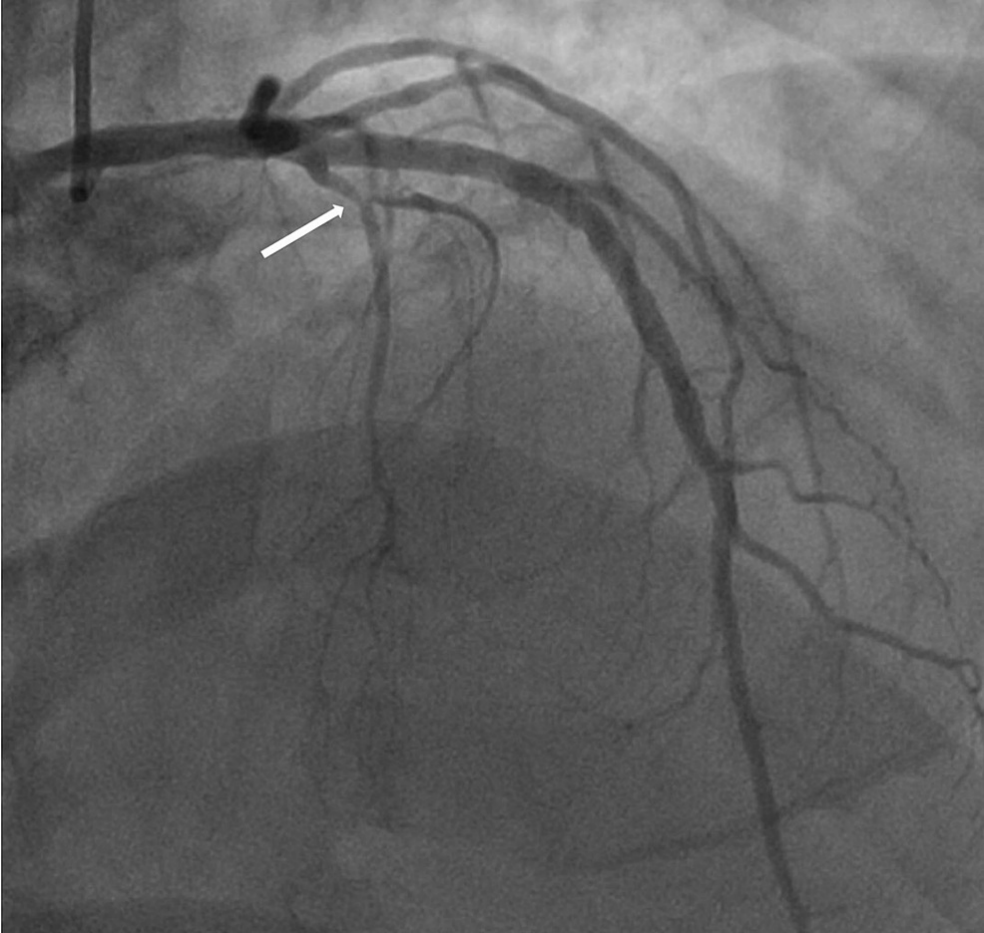
Invasive coronary angiography in the right anterior oblique (RAO) projection showing non-obstructive coronary artery disease in the first septal perforator branch (arrow).

Testing for respiratory viruses, including severe acute respiratory syndrome coronavirus 2 (SARS-CoV-2), adenovirus, influenza A, parainfluenzae, and respiratory syncytial virus were all negative. Chest X-ray was negative for any acute cardiopulmonary process. Overnight, troponin-I up-trended, peaking at 4.96 ng/mL 8 hours after hospitalization. Thereafter, he underwent a coronary angiogram that showed non-obstructive coronary artery disease in the first septal perforator branch (Figure 2). 

**Figure 3:**
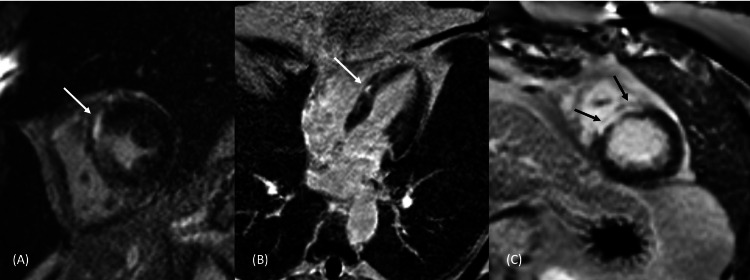
Short-axis phase-sensitive inversion recovery (PSIR) delayed enhanced images (A, C) show mid-myocardial enhancement at the mid-ventricular and apical levels of the myocardium respectively, and a similar enhancement pattern is illustrated in the delayed enhanced horizontal long-axis view (B) in the same patient.

The patient reported spontaneous resolution of the chest pain within 24 hours of admission. Given the indeterminate etiology of the troponin-I elevation, a CMRI was performed, which showed a reduced left ventricular ejection fraction of 44%, with myocardial and epicardial enhancement along the anterior septum in the mid-ventricular level extending to base, sparing the subendocardium (Figure 3). These findings were found to be consistent with myocarditis. The patient had received his second dose of the Pfizer-BioNTech vaccine three months prior to presentation, and his acute myocarditis was ascribed to a late myocarditis presentation after exposure to the COVID-19 vaccine.

## Discussion

Myocarditis has been traditionally described as an inflammation of the myocardial wall, which can lead to a variety of structural and conduction abnormalities [1,4]. Post-vaccination myocarditis has been previously described after smallpox and influenza vaccines [1,4,5,7]. The BNT162b2 and mRNA-1273 vaccines have been shown to confer a protection rate of 95% and 94.1% against symptomatic COVID-19 in individuals aged 16 or older, respectively [2]. These vaccines have an excellent safety profile, with the common adverse effects being arm pain (88.04%), generalized weakness (58.89%), headache (45.48%), chills (36.60%), etc. Only 1.12 % of the study cohort had chest pain post-COVID-19 vaccine [2]. Vaccine-induced myocarditis has been recently described as an adverse effect of these mRNA vaccines. Among the reported cases of myocarditis post-COVID-19 mRNA vaccination, 55% were seen with mRNA-1273 vaccine and 45% were seen with BNT162b2 vaccine [1,3-9]. Based on a review of the literature, it has been seen more commonly in males younger than 40 years of age [1,3-9]. There have been isolated case reports of elderly individuals presenting with myocarditis following COVID-19 vaccination [1]. However, most had presented shortly after receiving the second dose, except for one patient who presented after the first dose of the vaccine [8]. Presentation within four days is common, though there has been a patient presenting 15 days after vaccination [6]. Interestingly, our patient had myocarditis three months after his second dose of the vaccine. 

Among the cases described, all of the patients had variable ECG changes at presentation. CMRI changes suggestive of myocarditis were present in all the patients [1,3-9]. The ECG and CMRI changes are summarized in Table 1. Vaccine-induced myocarditis has a relatively benign course, with most patients making a full recovery within a week. Our patient’s clinical course was significant for the resolution of symptoms within 48 hours of presentation and was discharged on a beta-blocker.

**Table 1:** Summary of cases of post COVID vaccine myocarditis. CMRI: cardiac magnetic resonance imaging; NSAID: nonsteroidal anti-inflammatory drug

Study, No of patients (n)	Comorbidities	Associated vaccine	Age (years)	Timing of presentation (post-second dose of vaccine)	Presenting symptoms	ECG	Tn +ive	CMRI changes	Treatment	Prognosis
Habib et al. [1] N=1	Former smoker	BNT162b2	37/M smoker	36 hours	Squeezing chest pain	Mild ST elevation in anterior leads.	+	Early and late faint subepicardial enhancement of the basal lateral wall.	DAPT, metoprolol.	6-day hospital stay, patient was asymptomatic at discharge.
Jay Montgomery et al. [3] N=23	Healthy	7 received BNT162b2-mRNA; 16 received mRNA-1273 vaccine	20-52/M	Within 96 hours	Sharp chest pain	ST elevations, T wave inversion and nonspecific ST changes were seen in 83% of patients.	+	n=8, which showed SE late gadolinium enhancement and/or focal myocardial edema	Rapid recovery was seen in all patients.	Symptoms resolved in 1 week for 16 patients. Follow-up data wasn’t available for the rest of the seven patients.
Mansour et al. [4] N=2	Healthy	mRNA -1273 for both patients.	21,25 M:F=1:1	Within 48 hours	Sharp retrosternal chest pain	Mild ST elevations and PR depression	+	SE late gadolinium enhancement was seen in both patients.	Beta blocker	Symptoms resolved within 24 hours, no data on long-term follow-up.
Kim et al. [5] N=4	3 males and 1 female	2 received mRNA -1273, 2 received BNT162b2 vaccine	36(M), 23(M), 70(F), 24(M)	Within 2-5 days	Severe chest pain	ST elevations	+	Regional dysfunction, late gadolinium enhancement, and elevated native T1 and T2.	NSAIDs +/- colchicine	Patients discharged within 2-4 days, none requiring rehospitalization.
Muthukumar et al. [6] N=1	HTN, HPLD, OSA.	mRNA 1273 vaccine	52(M)	Within 72 hours	Mid sternal chest discomfort	Left axis deviation, No ST-T changes.	+	Mid myocardial and SE linear and nodular late gadolinium enhancement (LGE) in the inferoseptal, inferolateral, anterolateral, and apical walls.	Low dose Lisinopril and carvedilol. No immunosuppressive or anti-inflammatory medications.	4-day hospital course. No rehospitalization in the following three months.
Ammirati et al. [7] N=1	Healthy	BNT162b2 vaccine	56(M)	Within 72 hours	Acute chest pain	Minimal ST elevation in precordial leads with peaked T waves	+	Focal SE -intramyocardial (non-ischemic pattern) late gadolinium enhancement (LGE) involving the basal and apical segments of the inferolateral wall	Supportive therapy	7-day uncomplicated hospital course.
Abu Mouch et al. [8] N=7	Healthy	BNT162b2 vaccine	Median age: 23(M)	Six patients within 24-72 hours, one patient presented after 15 days.	Chest pain	ST elevation noted in all patients	+	CMR was done in all patients, which was suggestive of myocarditis(myocardial edema and late gadolinium enhancement)	NSAID + Colchicine	Mild hospital course, data not available on long-term follow-up.
Albert et al. [9] N=1	Healthy	mRNA-1273	24(M)	4 days	Chest discomfort	Sinus rhythm, without any ST changes	+	Patchy mid-myocardial and epicardial delayed gadolinium enhancement, with superimposed edema	Unclear	Unclear

## Conclusions

The present case report describes a late manifestation of acute myocarditis in an elderly male patient occurring three months after receiving the second dose of an mRNA COVID-19 vaccine. Although causality could not be established, there was no other identifiable cause to explain the occurrence of acute myocarditis. The present case report provides new evidence regarding possible subacute to late-onset of myocarditis post-COVID-19 vaccine. Additionally, given the early symptom resolution, it highlights the favorable outcome in this newly described clinical entity.
